# The Effect of Alcohols on Red Blood Cell Mechanical Properties and Membrane Fluidity Depends on Their Molecular Size

**DOI:** 10.1371/journal.pone.0076579

**Published:** 2013-09-23

**Authors:** Melda Sonmez, Huseyin Yavuz Ince, Ozlem Yalcin, Vladimir Ajdžanović, Ivan Spasojević, Herbert J. Meiselman, Oguz K. Baskurt

**Affiliations:** 1 Koc University, School of Medicine, Istanbul, Turkey; 2 University of Belgrade, Institute for Biological Research “Siniša Stanković”, Belgrade, Serbia; 3 University of Belgrade, Institute for Multidisciplinary Research, Belgrade, Serbia; 4 Department of Physiology and Biophysics, Keck School of Medicine, Los Angeles, California, United States of America; University of North Carolina at Chapel Hill, United States of America

## Abstract

The role of membrane fluidity in determining red blood cell (RBC) deformability has been suggested by a number of studies. The present investigation evaluated alterations of RBC membrane fluidity, deformability and stability in the presence of four linear alcohols (methanol, ethanol, propanol and butanol) using ektacytometry and electron paramagnetic resonance (EPR) spectroscopy. All alcohols had a biphasic effect on deformability such that it increased then decreased with increasing concentration; the critical concentration for reversal was an inverse function of molecular size. EPR results showed biphasic changes of near-surface fluidity (i.e., increase then decrease) and a decreased fluidity of the lipid core; rank order of effectiveness was butanol > propanol > ethanol > methanol, with a significant correlation between near-surface fluidity and deformability (r = 0.697; p<0.01). The presence of alcohol enhanced the impairment of RBC deformability caused by subjecting cells to 100 Pa shear stress for 300 s, with significant differences from control being observed at higher concentrations of all four alcohols. The level of hemolysis was dependent on molecular size and concentration, whereas echinocytic shape transformation (i.e., biconcave disc to crenated morphology) was observed only for ethanol and propanol. These results are in accordance with available data obtained on model membranes. They document the presence of mechanical links between RBC deformability and near-surface membrane fluidity, chain length-dependence of the ability of alcohols to alter RBC mechanical behavior, and the biphasic response of RBC deformability and near-surface membrane fluidity to increasing alcohol concentrations.

## Introduction

The physicochemical properties of membranes are of critical importance for many fundamental functions of cells, with the specific effects of these properties determined by complex cellular mechanisms [1]. Mammalian red blood cells (RBC) have a relatively simple structure (i.e., membrane surrounding a hemoglobin solution with no internal structures), and thus the properties and functions of the cell membrane are of primary importance. RBC have unique mechanical properties, which are crucial for the maintenance of *in vivo* blood flow [2]: when exposed to physiological levels of shear stress (SS), RBC are able to deform and change their shape. This ability to respond to SS, known as cellular deformability, enables these ~8 micron-diameter cells to pass through vessels with diameter as small as 3 microns, thereby significantly contributing to normal blood flow resistance at the microcirculatory level [3]. Further, the high deformability of RBC provides them with mechanical stability, tending to protect them from fragmentation and hemolysis under hemodynamic shear forces [4]. Decreased deformability and decreased stability of RBC might result in: (i) impaired blood flow, which may lead to ischemia and hypertension; (ii) endothelial dysfunction; (iii) chronic hypercoagulable state, which may lead to thromboembolic events [5–7].

It is well known that RBC deformability and stability, which represent parameters having direct *in vivo* implications, are determined by the mechanical and structural characteristics of the cytoskeleton and lipid bilayer [3,8,9]. A positive correlation between RBC deformability and membrane fluidity has been previously suggested, but to date this concept has not been examined in a coherent and detailed manner [10–13]. In addition, the relation between deformability and the fluidity at different membrane depths (e.g. surface, hydrophobic core) has never been studied.

Alcohols interact with lipid bilayers, with the -OH group positioned in the bilayer interfacial region and with the hydrocarbon chains facing the hydrophobic core of the bilayer. The relevant characteristics of alcohols include: (i) A simple structure with hydrophilic and lipophilic moieties; (ii) The size of their lipophilic moiety and their partition coefficient into the bilayer gradually increase with increasing chain length; (iii) They insert deeper into membrane layers with increasing concentration and can penetrate into the hydrophobic core [14,15]; (iv) Their penetration into the lipid bilayer increases with the length of the alcohol’s hydrophobic chain [16]; (v) They appear to modify both the deformability and fluidity of RBC.

The aims of the present study were (i) to examine the effects of small alcohols on RBC mechanics and membrane stability; (ii) to acquire new information regarding the interactions of small alcohols with cellular membranes using RBC as a model; (iii) to determine the potential relationship between RBC deformability and membrane fluidity using laser diffraction ektacytometery and electron paramagnetic resonance (EPR) with two spin-labels -5-DS and 16-DS that provide fluidity information of the near-surface membrane layer [17,18] and the deeper membrane layer [19]. Four members of a homologous series of primary alcohols (i.e., methanol, ethanol, 1-propanol, 1-butanol) were selected for study in order to explore the effects of hydrophobic chain length; alcohol concentrations were ≥ 0.5% and thus above physiological levels.

## Materials and Methods

### Blood samples

Venous blood samples were obtained from healthy volunteers, aged between 19 to 57 years, using vacuum tubes with ethylendiaminetetraacetic acid (EDTA, 1.8 mg/mL). A tourniquet was applied to the upper arm during sampling from antecubital vein and sampling was completed within 90 s following the tourniquet application [20]. All procedures related to the use of human blood in this study are in conformity with the recommendation provided in The Code of Ethics of the World Medical Association (Declaration of Helsinki) for experiments involving humans. The protocols were approved by the Institutional Review Board (IRB) of Koc University, Istanbul, Turkey and Ethics Committee of Institute for Biological Research "Siniša Stanković", Belgrade, Serbia. The subjects provided verbal consent, following the explanation of the blood sampling procedure and the protocols briefly; a written consent was not seen necessary by the IRB and Ethics Committee as the procedure was accepted not to involve more than minimal risk. The information related to the volunteers are kept in a separate data file, which is not directly linked to the experimental data files. Experiments were completed within 4 hours following blood sampling.

### Preparation of RBC suspensions containing alcohols

Whole blood was diluted 1:200 in a viscous medium containing alcohol at various concentrations for ektacytometry. Methanol, ethanol, propanol and butanol were added to a 6% solution of high-molecular weight polyvinylpyralidone (PVP, 360 kDa) prepared in isotonic-phosphate buffered saline (PBS, pH = 7.4) at concentrations presented in Table 1; these concentrations were selected based on preliminary studies. The viscosities of the PVP solutions were measured at 37°C following the addition of alcohols using a Wells-Brookfield cone-plate rotational viscometer. The viscosity of the PVP solution was 23.0 mPa·s without added alcohol but was slightly increased by the addition of ethanol, propanol and butanol, with the increase being a function of both alcohol molecular size and concentration; no change of viscosity was observed with methanol at any concentration (Table 1). Measured viscosity values were used to calculate the rotational speed (i.e., shear rate) of the ektacytometer that are needed to achieve a given SS.

**Table 1:** Concentrations of methanol, ethanol, propanol and butanol used and the viscosity of PVP solutions containing these alcohols (measured at 37°C).

**Methanol**			**Ethanol**			**Propanol**			**Butanol**		
**Concent.**		**Visc.**	**Concent.**		**Visc.**	**Concent.**		**Visc.**	**Concent.**		**Visc.**
(%)	(M)	(mPa·s)	(%)	(M)	(mPa·s)	(%)	(M)	(mPa·s)	(%)	(M)	(mPa·s)
0	0	23.0	0	0	23.0	0	0	23.0	0	0	23.0
0.5	0.123	23.0	0.5	0.086	23.2	0.5	0.067	23.2	0.5	0.055	23.5
1	0.247	23.0	1	0.171	23.4	1	0.133	23.5	1	0.109	23.8
2	0.494	23.0	2	0.343	23.8	2	0.266	23.8	2	0.219	24.2
4	0.988	23.0	4	0.687	24.7	4	0.532	25.1	-	-	-
5	1.234	23.0	5	0.859	25.1	5	0.665	26.4	-	-	-
-	-	-	7	1.202	25.5	7	0.932	27.2	-	-	-

### Assessment of RBC deformability

RBC elongation indexes (EI) were determined as a function of applied SS using a laser diffraction ektacytometer (LORCA MaxSis, Mechatronics, Hoorn, The Netherlands). The system uses a co-axial cylindrical shearing system; both the inner and outer cylinders are made of transparent material with a gap of ~350 µm between them. The outer cylinder rotates at a calculated rotational speed to generate the desired SS in the sample introduced into the gap. The suspending medium viscosity is also a determinant of SS and hence the viscosity values shown in Table 1 were utilized. A laser beam (670 nm wavelength) is projected perpendicular to the rotation axis and the diffraction pattern generated by RBC in the gap on the opposite side is captured by a CCD camera and analyzed by microcomputer that also controls the rotation of the outer cylinder. EI were calculated during the application of 10 steps of SS, in the range of 0.3 to 50 Pa, by using the long and short axes of the elliptical diffraction patterns (a and b, respectively) as EI= (a-b)/(a+b); the data are expressed as EI-SS curves. Additionally, the maximum EI (EI_max_) and the shear stress required to cause one-half of this maximum (SS_1/2_) were determined using the Lineweaver-Burk approach and non-linear curve fitting [21]. The SS_1/2_/EI_max_ ratio was calculated as a normalized measure of SS_1/2_ [22]; SS_1/2_/EI_max_ is inversely related to RBC deformability such that a lower value indicates better deformability.

### Assessment of RBC mechanical stability

RBC, suspended in the viscous PVP solution, were subjected to constant 100 Pa SS using the ektacytometer. EI was continuously monitored for 300 s at this SS, and exhibited a characteristic time course. This EI-time course provides information about RBC fragmentation and related changes in deformability, thereby reflecting the influence of alcohols on membrane stability. The stability test was performed in the presence of methanol, ethanol, propanol and butanol in the suspending medium of RBC at concentrations listed in Table 1. Additionally, SS-EI curves were obtained after the application of 100 Pa SS for 300 s, and normalized SS_1/2_ values (SS_1/2_/EI_max_) were compared with those measured before the application of 100 Pa SS.

### EPR measurements of RBC membrane fluidity

RBC were spin-labeled as described previously [17,18]. In brief, RBC were isolated from fresh blood and washed three times with PBS by centrifugation at 3500g/10 min/4°C; the hematocrit of all samples was adjusted to ~40%. Ethanol solutions of spin-labels 5-DS and 16-DS (5- and 16-doxyl stearate, Sigma-Aldrich, St. Louis, MO, USA) were applied onto the walls of microtubes, with the amount of DS calculated to obtain an optimal spin label to membrane lipid ratio of approximately 1:100. After the ethanol had evaporated, RBC were added and gently mixed, and then methanol, ethanol, propanol or butanol were added to the RBC in the microtubes to achieve final molar concentrations shown in Table 1. RBC samples were placed in Teflon tubes with a wall thickness of 0.025 mm and an internal diameter of 0.6 mm (Zeus Industries, Raritan, NJ, USA) and inserted into quartz capillaries. EPR spectra were recorded at 20°C, 2 min after the addition of alcohols, using a Varian E104-A EPR spectrometer (Palo Alto, CA, USA) operating at X-band (9.1 GHz) with the following settings: modulation amplitude, 2 G; modulation frequency, 100 kHz; microwave power, 20 mW; scan range, 100 G; scan time, 4 min; time constant, 0.25 s. Spectra were recorded and analyzed using EW software (Scientific Software Inc., Bloomington, IL, USA), and the order parameter (S) of 5-DS-labeled RBC, which is reciprocally proportional to fluidity, was calculated as described previously [17,18]. 5-DS has a paramagnetic group at C5 in the fatty acid chain which enables the evaluation of fluidity of the layer near the membrane surface, while 16-DS has a paramagnetic group much deeper in the membrane, at C16 of the fatty acid chain, which therefore enables evaluation of membrane fluidity in the membrane’s hydrophobic core [23]. For 16-DS, it is more appropriate to calculate rotational correlation times (τ) rather than the order parameter (S), where τ is also reciprocally proportional to fluidity. Rotational correlation times were calculated as described previously [19,24]. All experiments were performed at least in quadruplicate.

### Assessment of hemolysis

Blood was diluted 1:200 in PBS containing methanol, ethanol, propanol or butanol at concentrations listed in Table 1; 1: 200 dilution samples without alcohol were used as a control and for the determination of total hemoglobin. These dilute RBC suspensions were incubated at room temperature for 30 min then, with the exception of the total hemoglobin sample, centrifuged at 400g/5 min/25°C. An aliquot of supernatant from each centrifuged sample and the same volume of the total hemoglobin sample were mixed with 10-fold concentrated Drabkin’s reagent containing 5% Triton X-100. The absorbance (Abs) was read at 540 nm. Hemolysis percentages for each alcohol and each concentration were calculated as follows: Hemolysis (%) = (Abs_sample_-Abs_control_) / (Abs_total hemoglobin_) x 100.

### Assessment of RBC morphology

RBC morphology was assessed in freshly prepared, wet-mount preparations. Briefly, dilute RBC (1:200 dilution of blood) in PBS containing various alcohols at the concentrations used in the experiments were transferred onto glass microscope slides having a silicone grease well of ~1 mm depth, and covered with a coverslip. RBC were observed approximately mid-way between the slide and coverslip. This method allows observation of RBC in the suspension away from glass surfaces, therefore minimizing the crenation artifact caused by surface effects. The RBC were not stained. Micrographs were obtained using a light microscope (Zeiss Axio Scope A1 with Axiocam Icc3, Carl Zeiss MicroImaging GmbH, Göttingen, Germany).

### Data presentation and statistics

Data are presented as mean ± standard error (SE), except for S and τ which are presented as mean ± standard deviation (SD). Two-way ANOVA followed by "Bonferroni post test" were used for comparison of EI-SS curves obtained in the presence of various alcohols. RBC deformability parameters SS_1/2_, EI_max_ and the SS_1/2_/EI_max_ ratio, S and τ, as well as percent hemolysis were compared with one-way ANOVA. Non-linear curve fitting using a Lineweaver-Burk approach was performed using Graphpad 4.0 (GraphPad Software, La Jolla, CA, USA).

## Results

### Effect of alcohols on RBC deformability

**Figure 1:**
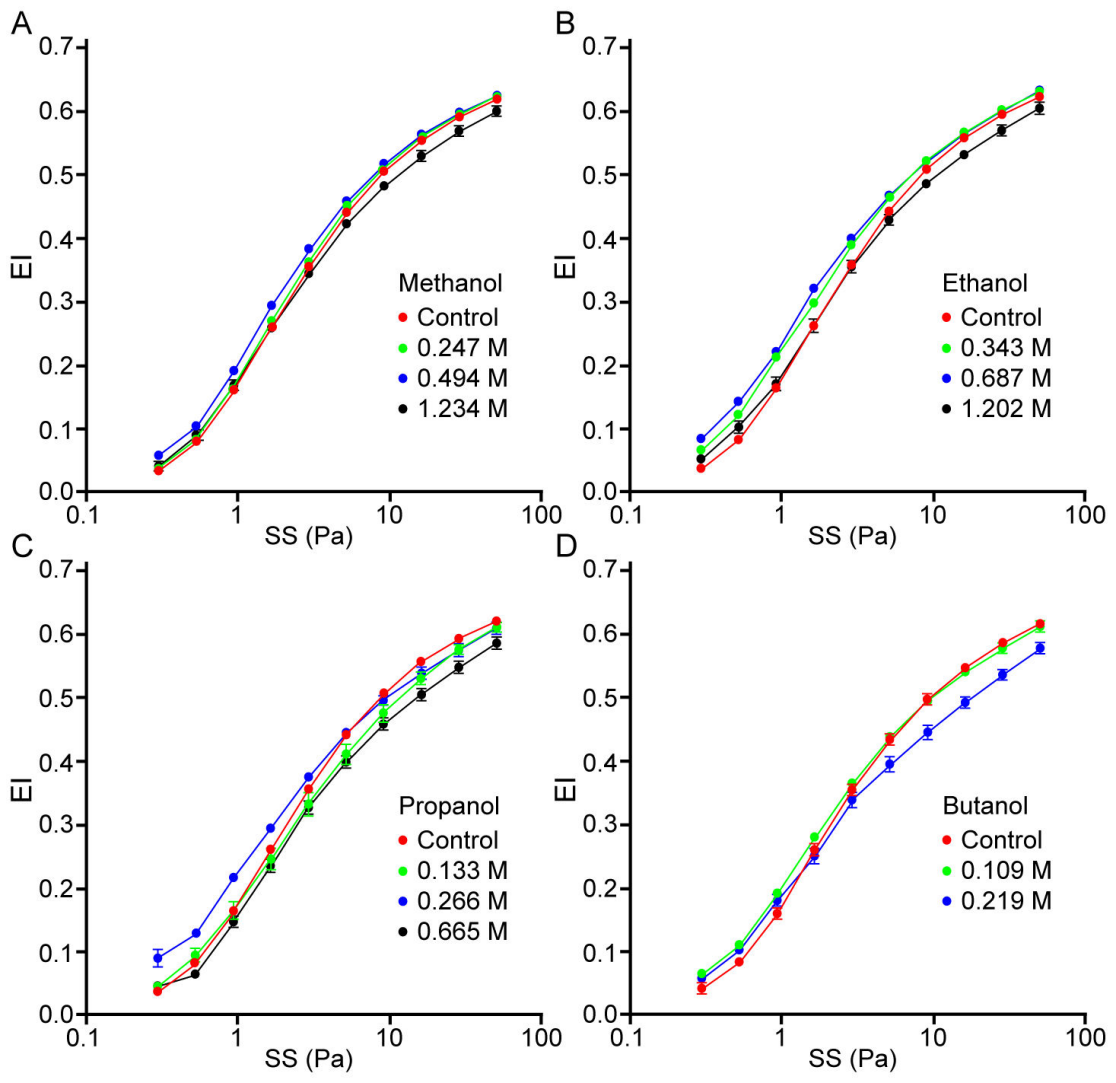
Elongation index (EI)-shear stress (SS) curves for RBC in suspending medium containing the four alcohols at different concentrations. (**A**) Methanol. (**B**) Ethanol. (**C**) Propanol. (**D**) Butanol. The data are means ± SE from 10 experiments on blood samples from different donors. Note that error bars often lie within the symbols and therefore are not visible. The curves representing the EI-SS relations for RBC suspensions containing different concentrations of alcohols were significantly different from each other for all for alcohols as determined by two-way ANOVA (P<0.001).

Figure 1 presents EI-SS curves for RBC suspensions containing methanol, ethanol, propanol or butanol at various concentrations. For all four alcohols, EI measured at SS in the low to mid-range (i.e., 0.3–9 Pa) increased with alcohol concentration, reaching a maximum at a specific concentration that depended on the molecular size of the alcohol. The increase of EI above control was statistically significant (P<0.05) for 0.494 M methanol in the SS range of 0.3 to 5.15 Pa (Figure 1A). Significant increase was observed in the same SS range with ethanol at 0.343 M and 0.687 M (P<0.001; Figure 1B), and for propanol at 0.266 M (P<0.001; Figure 1C). The only significant improvement in EI with butanol was detected at 0.109 M with 0.94 Pa SS (P<0.05; Figure 1D). Further increases of alcohol concentration above a critical level specific for each alcohol resulted in significantly reduced EI, especially at higher SS (i.e., 9–50 Pa); the critical concentrations at which EI started to decrease were 0.219 M for butanol, 0.665 for propanol, 1.202 M for ethanol, and 1.234 M for methanol.

**Figure 2:**
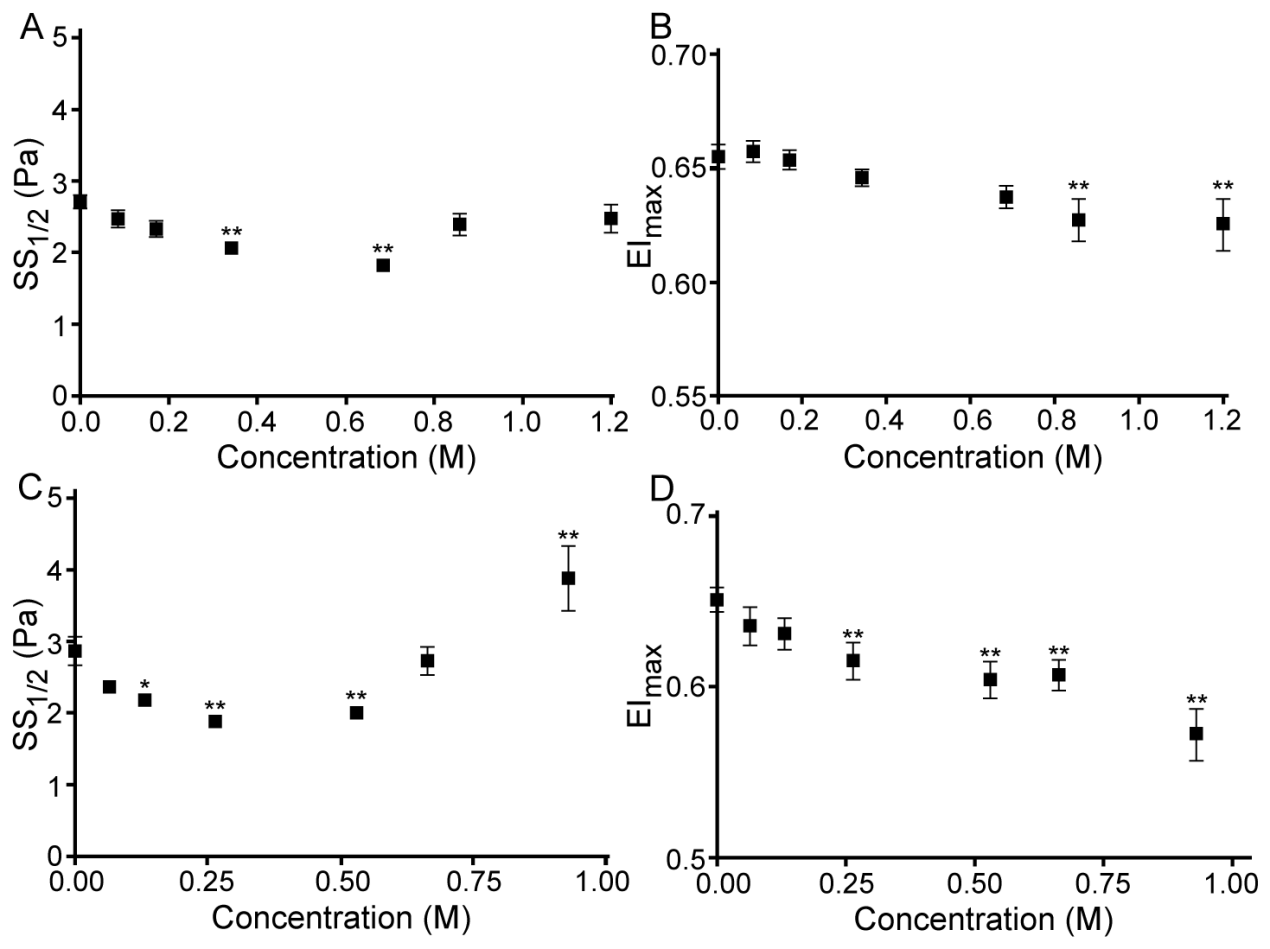
Half-maximal shear stress (SS_1/2_) and maximum elongation index (EI_max_) calculated by Lineweaver-Burk approach. The curves presented in Figure 1 were used in calculus. (**A**) SS_1/2_ for RBC exposed to ethanol; (**B**) EI_max_ for RBC exposed to ethanol; (**C**) SS_1/2_ for RBC exposed to propanol; (**D**) EI_max_ for RBC exposed to propanol. The data are means ± SE from 10 experiments on blood samples from different donors. Difference from control without alcohol were tested by one-way ANOVA: * P<0.05; ** P<0.01.

**Figure 3:**
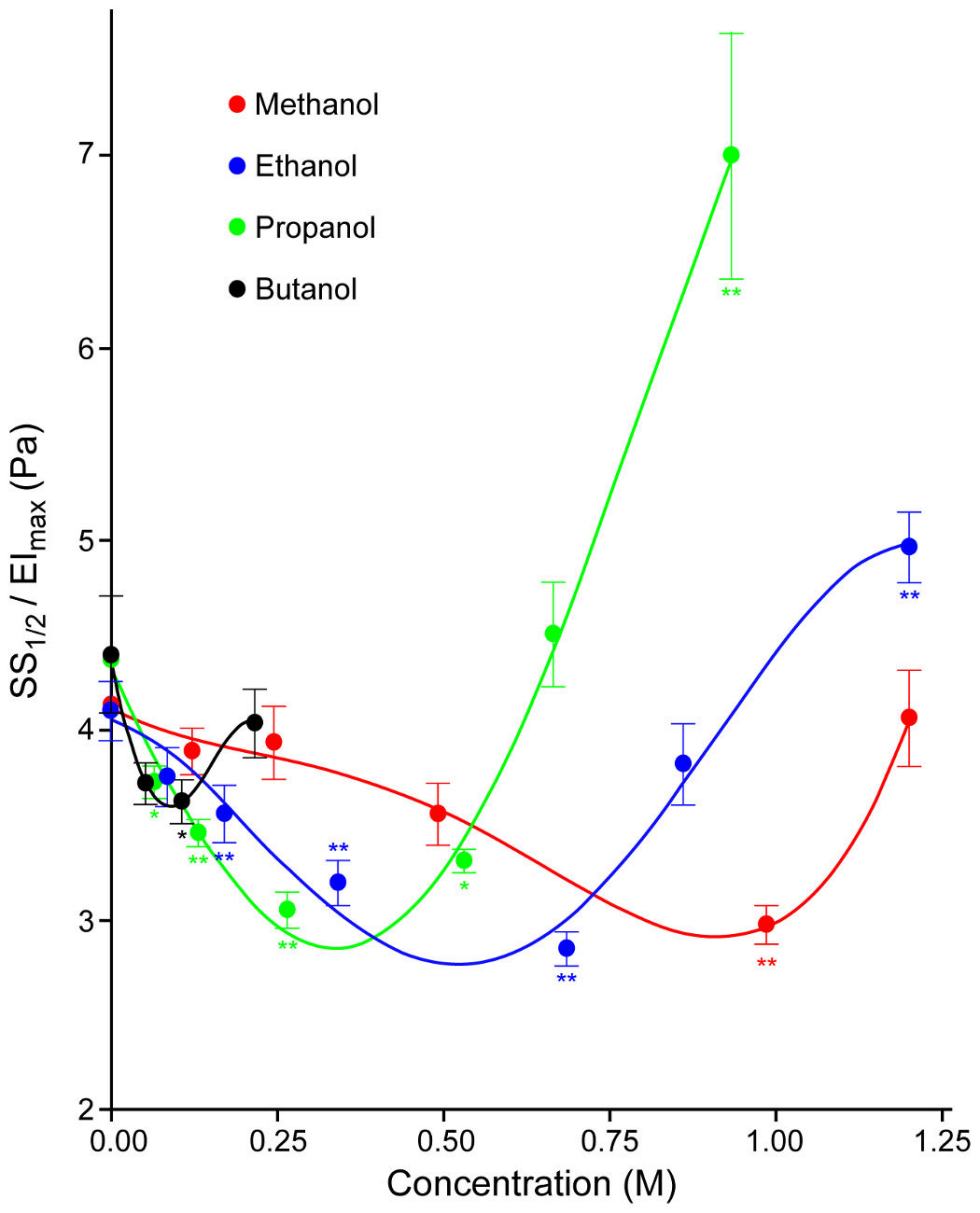
Effect of alcohol concentration on SS_1/2_/EI_max_ parameters. The effects were determined using the EI-SS curves presented in Figure 1. SS_1/2_/EI_max_ is inversely proportional to RBC deformability. The data are means ± SE from 10 experiments on blood samples from different donors. Statistical significance of the differences in comparison to control (no alcohol) are: * P<0.05 and ** P<0.01, as tested by one-way ANOVA.

**Figure 4:**
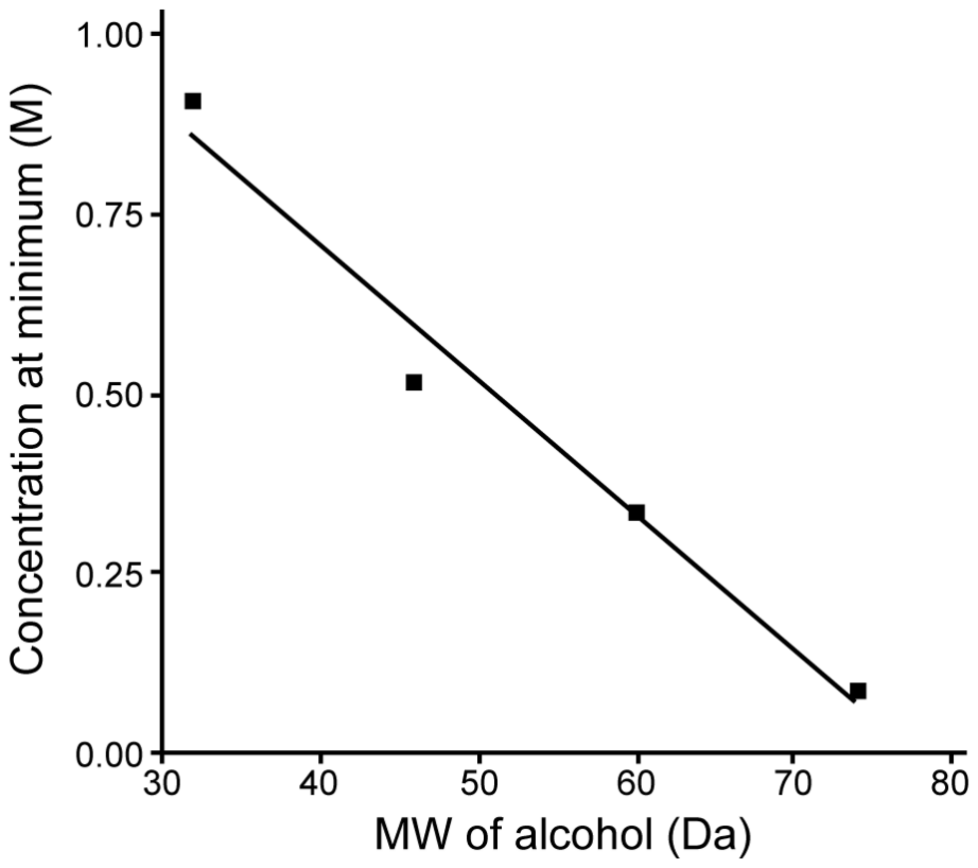
Alcohol concentrations at which the SS_1/2_/EI_max_ parameter reached a minimum (i.e., maximal RBC deformability). The minimal concentrations were determined using the fitted curves presented in Figure 3.

This pattern can also be clearly recognized for the SS_1/2_ parameter obtained from EI-SS curves (see Figure 2 for ethanol and propanol). SS_1/2_ values decreased significantly at 0.343 and 0.687 M of ethanol (P<0.01), but approached control values at higher concentrations (Figure 2A); a similar pattern was found for other alcohols. For example, the exposure of RBC to propanol resulted in a biphasic alteration of SS_1/2_; a decrease of SS_1/2_ was significant between 0.133 and 0.532 M (P<0.05 and P<0.01, respectively), while the SS_1/2_ for RBC suspensions at 0.932 M of propanol were significantly increased compared to control, thus indicating an impairment of deformability at this highest propanol concentration (Figure 2C). Figure 2B and 2D demonstrate that EI_max_ values are also altered significantly by alcohols. Given that both SS_1/2_ and EI_max_ are affected, SS_1/2_/EI_max_ ratios were calculated in order to allow comparisons of the effects of the four alcohols at various concentrations [22]. The concentration-dependent influence of alcohols with different molecular weights is presented in Figure 3: the SS_1/2_/EI_max_ ratio reaches a minimum (i.e., the maximum of deformability) at a concentration specific to each alcohol, with reversal of the effects with further increase of concentration. It is important to note that butanol could only be tested at concentrations of up to 0.219 M, since higher concentrations caused significant hemolysis that precluded deformability measurements. However, the biphasic effect of butanol on deformability was clearly observed in the accessible concentration range (Figure 3). Note also that the concentration at which SS_1/2_/EI_max_ reaches a minimum is a linear inverse function of the molecular size of the alcohol (Figure 4).

### Effect of alcohols on RBC mechanical stability

**Figure 5:**
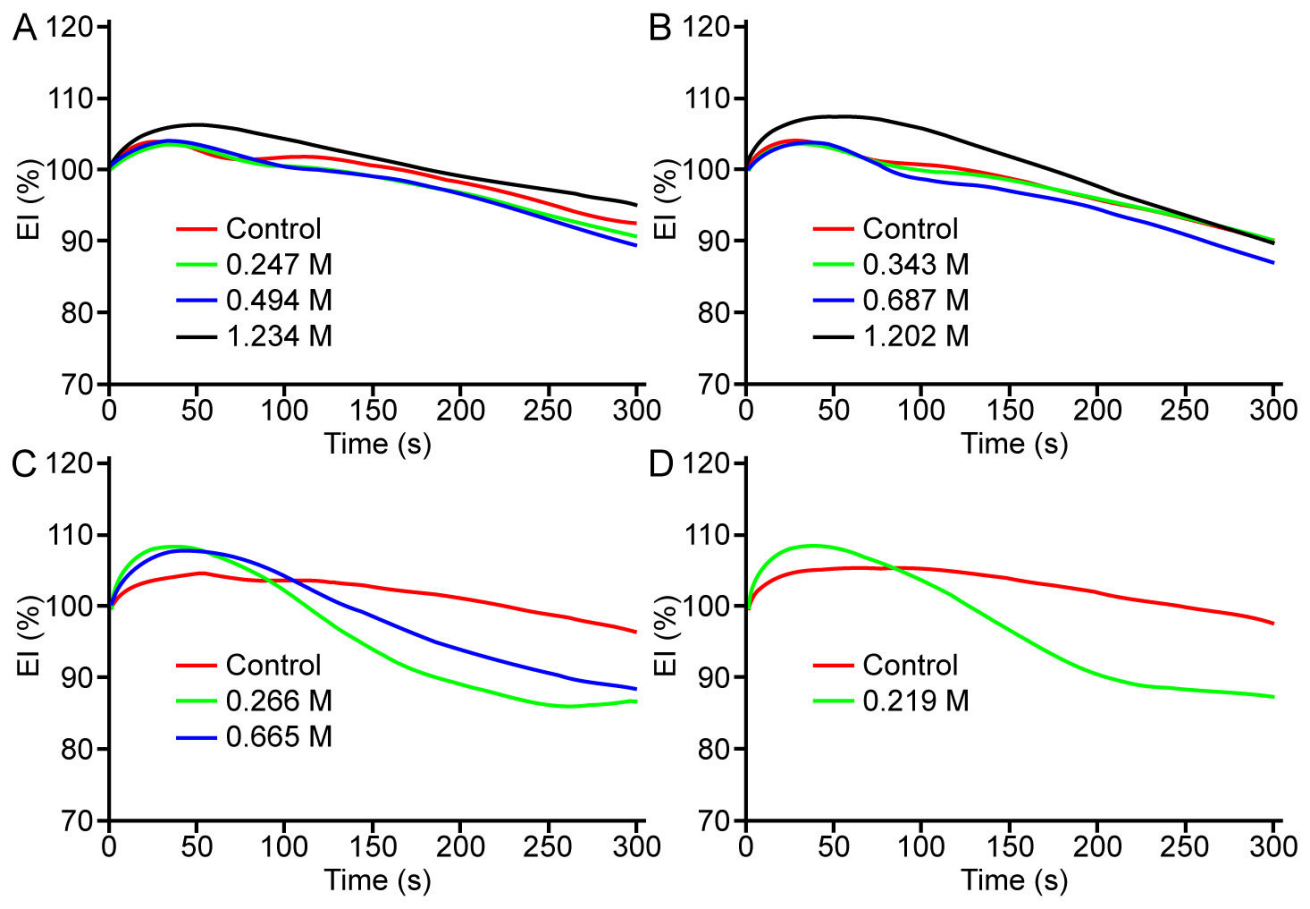
Effect of alcohol concentration on elongation indexes (EI) at 100 Pa SS during a 300 s period of shearing. (**A**) Methanol. (**B**) Ethanol. (**C**) Propanol. (**D**) Butanol. EI values are presented as % of the initial value. Each curve represents the mean from 10 experiments. Standard Error (SE) varied between 0.05 and 2.12, and showed increase with the duration of SS (error bars not shown for clarity). The curves were compared to control using two-way ANOVA, with significant differences (P<0.0001) observed for all four concentrations of methanol, ethanol at 0.687 and 1.202 M, both concentrations of propanol, and the single concentration of butanol.

Exposure of RBC suspensions to 100 Pa SS in the shearing system of the ektacytometer resulted in altered EI, which exhibited temporal changes (Figure 5). In general, for alcohol-free RBC suspensions, EI increased initially, reaching a maximum in the first 50 s, then approached a minimum between 50–100 s, followed by a small increase and a further decrease of EI, reaching a slightly lower EI at the end of 300 s of shearing. Inclusion of smaller alcohols (methanol and ethanol) at lower concentrations had no significant effects on the early parts of this time course, but affected the later parts of the EI-time curves. EI values measured at the end of the 300 s of exposure to 100 Pa were significantly lower in the presence of methanol at 0.247 M and ethanol at 0.687 M compared to control (Figure 5A and 5B). Propanol and butanol at comparable concentrations exhibited a different pattern (Figure 5C and 5D): (i) For propanol, EI increased initially, reaching a maximum within the first 60 s and then decreased, becoming significantly lower than control at 132 and 183 s for 0.266 and 0.665 M; (ii) For butanol, EI became significantly lower than initial control at 138 s for 0.219 M. A similar pattern (i.e., a progressive decrease of EI after the initial peak) was observed for methanol and ethanol only at extremely high concentrations (> 1.20 M); there was no significant decrease at ~300 s.

**Figure 6:**
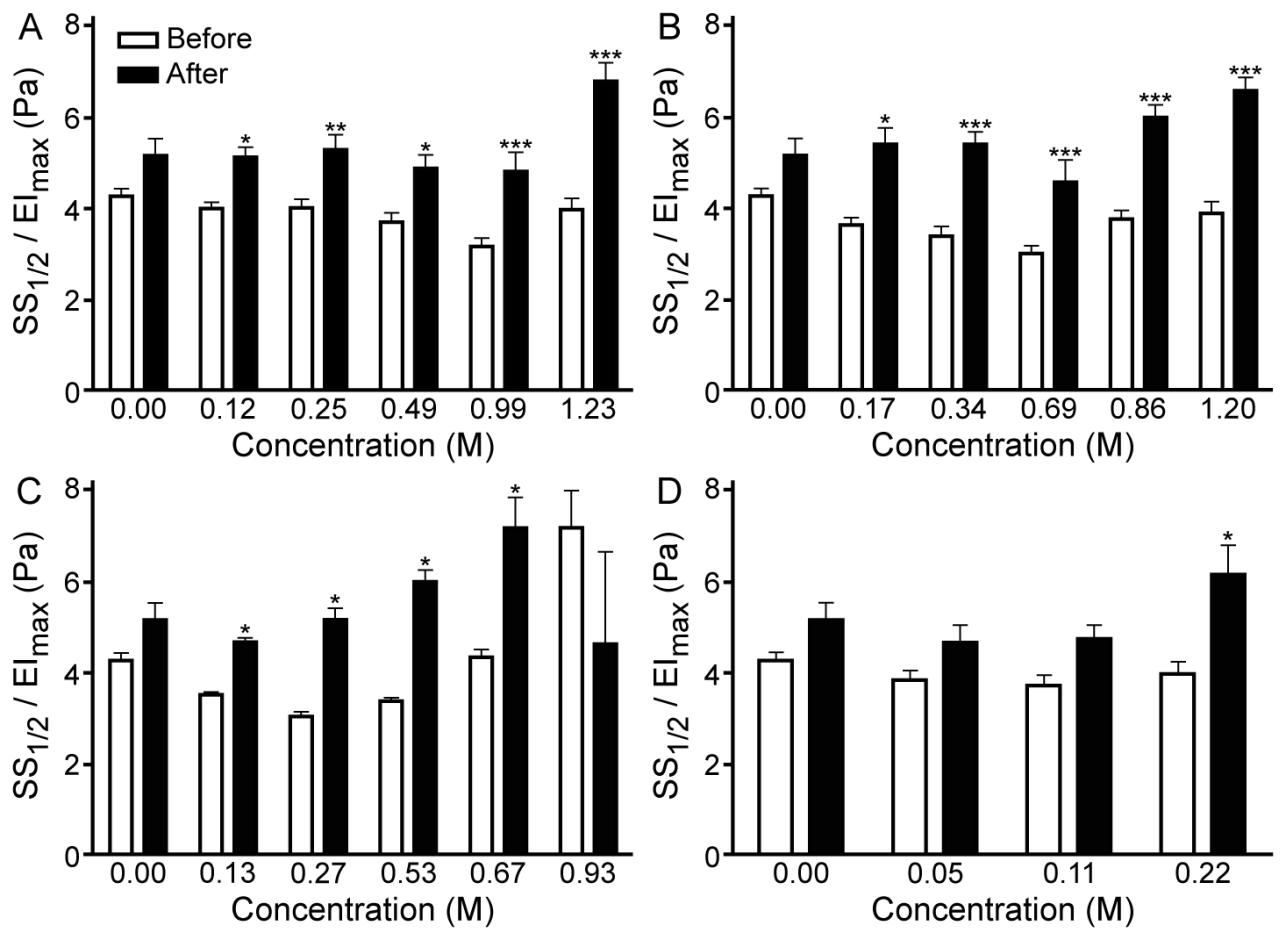
Changes of SS_1/2_/EI_max_ following the application of 100 Pa SS for 300 s. (**A**) Methanol. (**B**) Ethanol. (**C**) Propanol. (**D**) Butanol. Data are means + SE from 10 experiments on blood samples from different donors. Differences between values that were obtained before (white bars) and after (black bars) the exposure to 100 Pa SS, as determined by the means of two-way ANOVA: * P<0.05; ** P<0.01; *** P<0.001.

SS_1/2_/EI_max_ values, that were determined prior to and following shearing, indicated alterations of RBC mechanical stability that were modulated by the presence of alcohol (Figure 6). Following shearing at 100 Pa for 300 s, SS_1/2_/EI_max_ increased indicating an impairment of RBC deformability. Average differences of SS_1/2_/EI_max_ measured before and after the application of 100 Pa for 300 s are presented in Table 2; the alteration of SS_1/2_/EI_max_ increased with alcohol concentration. The effects on SS_1/2_/EI_max_ were especially evident at the higher concentrations of alcohol (at 1.234 M for methanol, ≥ 0.859 M for ethanol, 0.665 M for propanol, and 0.219 M for butanol), where the differences were significantly more pronounced than for control (Table 2). Higher concentrations of butanol resulted in significant hemolysis, and therefore were not employed in these experiments.

**Table 2:** Difference of SS_1/2_/EI_max_ parameters measured before and after the application of 100 Pa SS for 300 s.

**Methanol**		**Ethanol**		**Propanol**		**Butanol**	
**M**	**Difference**	**M**	**Difference**	**M**	**Difference**	**M**	**Difference**
0	0.92 ± 0.45	0	0.92 ± 0.45	0	0.48 ± 0.77	0	0.48 ± 0.77
0.123	1.12 ± 0.34	0.171	1.76 ± 0.34	0.133	1.17 ± 0.12	0.055	0.83 ± 0.17
0.247	1.27 ± 0.39	0.343	2.01 ± 0.33	0.266	2.14 ± 0.14	0.109	1.02 ± 0.25
0.494	1.20 ± 0.30	0.687	1.57 ± 0.52	0.532	2.62 ± 0.26	0.219	2.18 ± 0.82*
0.988	1.67 ± 0.50	0.859	2.22 ± 0.34*	0.665	2.83 ± 0.53*	-	-
1.234	2.81 ± 0.26**	1.202	2.69 ± 0.26**	0.932	-2.53 ± 2.46	-	-

M – molar concentration of each alcohol present during the test. Data are presented as mean + SE. Differences for each alcohol and concentration are compared to the difference observed in control suspensions not containing alcohol: *P<0.05; **P<0.01.

### Membrane fluidity alterations by alcohols

**Figure 7:**
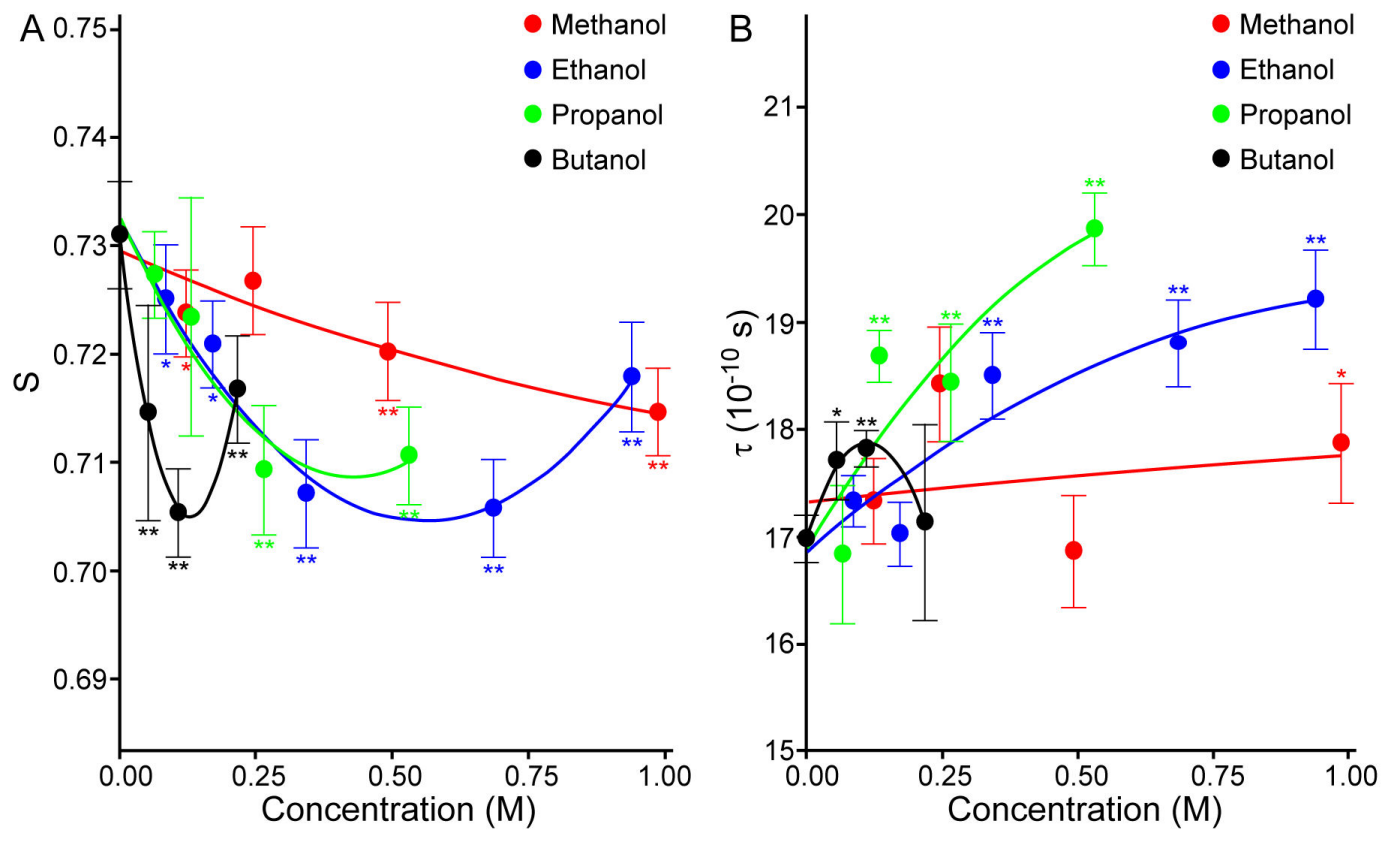
The effects of alcohols on RBC membrane fluidity at various concentrations as determined by EPR spectroscopy. (**A**) The order parameter (S) of 5-DS-labeled RBC. S for 2 M methanol was 0.729 ± 0.007 (not shown). (**B**) Rotational correlation time (τ) of 16-DS-labeled RBC. τ for 2 M methanol was 18.20 ± 0.88^**^ (10^-10^ s) (not shown). Data are mean ± SD from 4 experiments on blood samples from different donors. Statistically significant differences compared to control (no alcohol) using one-way ANOVA: * P<0.05, ** P<0.01.

EPR spectra of spin-labels 5-DS and 16-DS provide information about the fluidity of the near-surface membrane layer [17,18] and the deeper membrane layer [19], respectively. Both calculated parameters, S for 5-DS and τ for 16-DS, are inversely proportional to the fluidity of the specific membrane layer. Figure 7A shows dose-dependent changes of S in response to alcohols with different molecular weights. Note that at lower alcohol concentrations, fluidity gradually increases (i.e., S decreases), and that the downward slope and the concentrations at which the minimal S values occurred appear inversely related to the alcohol’s molecular weight. In other words, the rank order of the impact on membrane fluidity is butanol > propanol > ethanol > methanol. However, the minimal S value for propanol was only slightly and non-significantly different than ethanol. It should be noted that the S value does not appear to have reached a minimum for suspensions containing methanol in the range of concentrations shown in Figure 7A.

However, in additional experiments, the S parameter increased at higher concentrations of methanol (i.e., 2 M), clearly indicating a minimum at around 1 M. Therefore, similarly to other alcohols, the pattern of alterations of S for methanol mimics the biphasic pattern for SS_1/2_/EI_max_ (Figure 3).

The biphasic effects on membrane fluidity are clearly evident at higher alcohol concentrations. Interestingly, a comparison of concentration-dependent changes of deformability (Figure 3) and near-surface fluidity (Figure 7A) indicates very similar trends and the correlation between deformability and S was significant (Pearson r = 0.697; P<0.01). Additionally, the maximal RBC deformability (i.e., minimum SS_1/2_/EI_max_) and the highest membrane fluidity (i.e., minimum S parameter) were observed at similar concentrations, suggesting an association between these two cellular properties. Figure 7B presents the rotational correlation times. Inspection of these EPR data indicates that: (i) that methanol, ethanol, propanol and butanol all decrease the fluidity of the membrane hydrophobic core (i.e., increase τ); (ii) the concentrations at which the effects first become noticeable are inversely related to the alcohol’s molecular weight; (iii) at the highest concentration used, τ for butanol returned to control values.

### Hemolysis level

**Figure 8:**
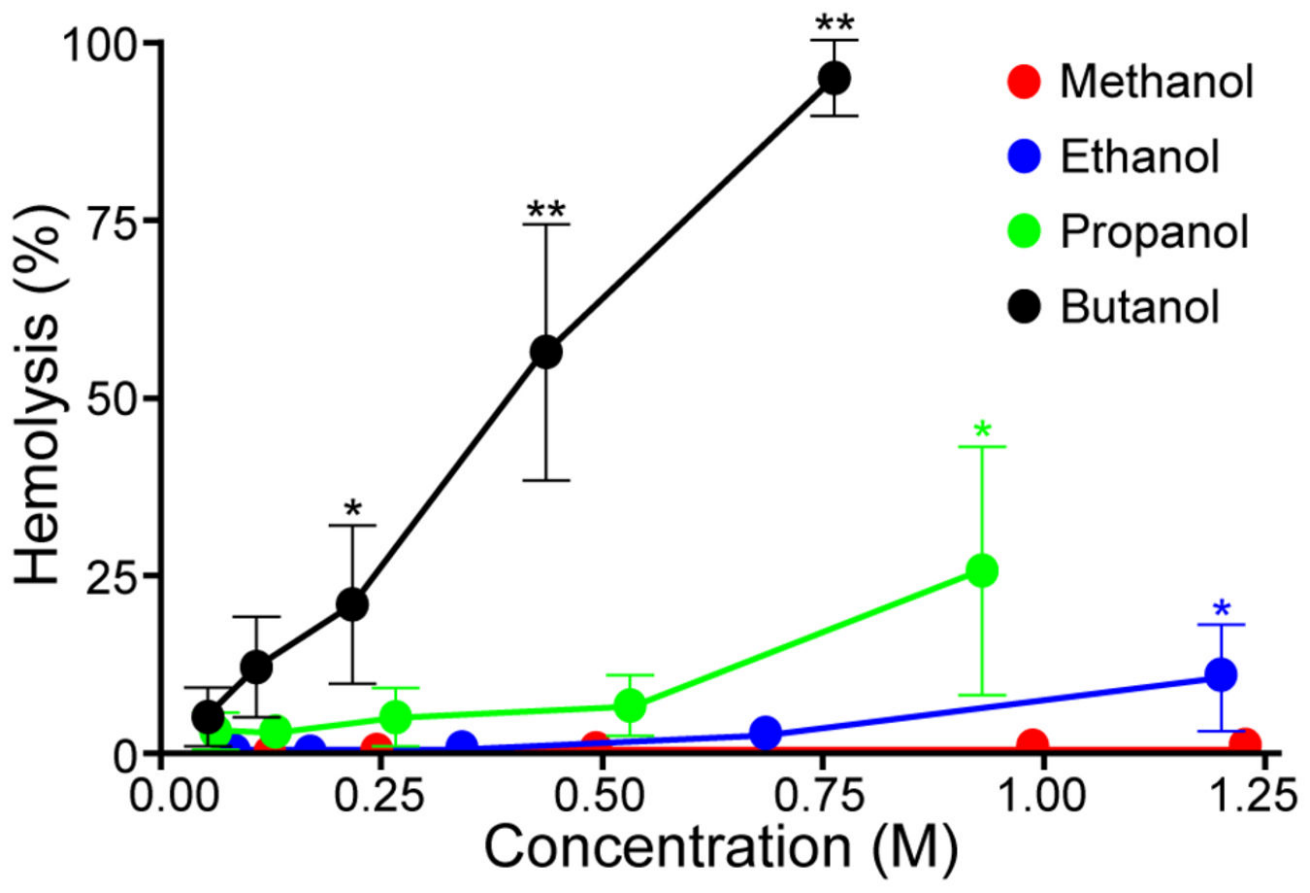
Hemolysis (%) plotted against alcohol concentration for four alcohols with different molecular sizes. Data are mean ± SE from 7 experiments on blood samples from different donors. Statistical significance from control (no alcohol) tested by one-way ANOVA: * P<0.05; ** P<0.01.

The level of hemolysis, which is provoked by the alcohols, depended on their molecular weight and concentration. While no hemolysis was observed for RBC exposed to methanol over the concentration range used in this study, hemolysis was observed with increasing concentrations of ethanol, propanol and butanol (Figure 8). Statistically significant increases were found for ethanol and propanol; hemolysis levels were 10.1±7.42% for ethanol at 1.202 M (P<0.05), 25.8±17.4% for propanol at 0.932 M (P<0.05), and 94.9±5.3% for butanol at 0.760 M (P<0.01).

### RBC morphology under the influence of alcohols

**Figure 9:**
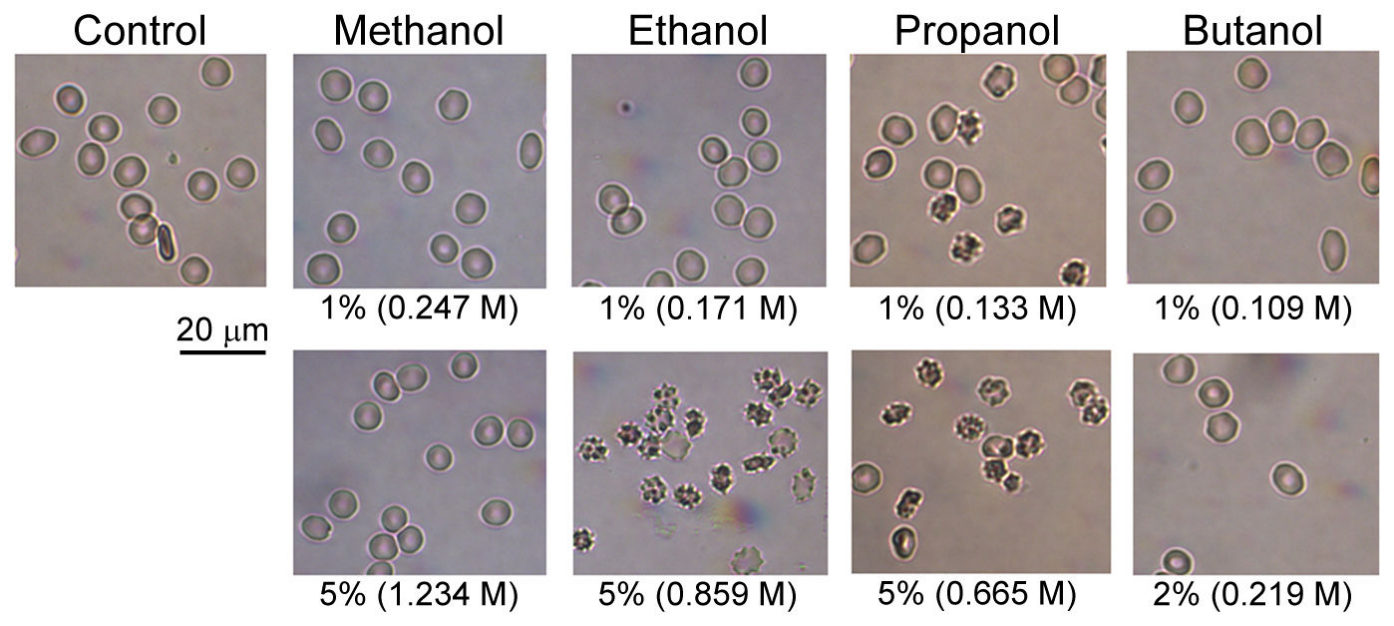
Light microscopy images of RBC in the presence of alcohols. The cells were observed in dilute (1:200), fresh, wet-mount, unstained preparations in which cell shape was unaffected by the "glass slide" effect (i.e., crenation due to alkaline conditions at glass surface).

Figure 9 presents the changes in RBC morphology that are provoked by alcohols. Echinocytosis was observed in the presence of ethanol at 0.859 M, with >80% of RBC being type II-III echinocytes [25], but was negligible in the suspensions with lower concentrations of ethanol. Morphological alteration of RBC started at lower concentrations for propanol, with ~40% of RBC being type I-III echinocytes at 0.133 M propanol and increased to >80% in suspensions containing 0.665 M propanol. Shape alterations were not observed in suspensions containing methanol and butanol in the range of concentrations used in this study.

## Discussion

The data obtained in the current study have shown three important aspects of the influence of alcohols with various molecular sizes on RBC mechanical properties: 1) Alcohols have biphasic effects on RBC deformability, wherein the deformability was increased at lower concentrations but the effects were diminished or even reversed at higher concentrations; 2) Alcohols at higher concentrations (i.e., 1.23 M for methanol, ≥ 0.86 M for ethanol, 0.67 M for propanol, and 0.22 M for butanol) reduced RBC mechanical stability as indicated by reduced deformability following the application of a non-physiological shear stress of 100 Pa; 3) Near-surface membrane fluidity showed a biphasic response to increasing concentrations of alcohols, with significant increase in fluidity in the lower concentration range, whereas alcohols caused a decrease of fluidity of the hydrophobic core. A positive correlation between RBC deformability and near-surface membrane fluidity was observed. The effects of alcohol on RBC deformability and membrane fluidity were positively associated with molecular weight.

Ethanol is the most widely investigated alcohol and its effects on RBC membrane and mechanical properties have been evaluated previously [15,26,27]; while corresponding data on the effects of other alcohols are missing in the literature. Various groups have previously reported improvement of RBC deformability by ethanol. Rabai and co-workers studied the effects of ethanol in a concentration range similar to the one applied here [28], and reported a progressive improvement of RBC deformability (i.e., decreased SS_1/2_ and SS_1/2_/EI_max_) in the presence of ethanol at concentrations between 0.5 to 5% (0.08 to 0.8 M), with a slight reversal of these parameters at 6% (~1 M). Ethanol has also been the subject of several clinically-oriented studies in which RBC mechanical properties were investigated [29–32]. The concentrations used in these clinical studies were 10–100 mM (i.e., 0.06-0.6%) and thus the results are not comparable with the data of Rabai [28] and the present study. However, improvement of RBC deformability has been reported even at these relatively low ethanol concentrations [29].

The influence of alcohols with molecular weights ranging between 32 to 74 Da on RBC deformability exhibited an interesting dose dependence, with an intermediate concentration having the maximum effect. This critical concentration (i.e., concentration for minimal SS_1/2_/EI_max_) for each alcohol was inversely proportional to its molecular weight: methanol 0.905 M; ethanol: 0.515 M; propanol: 0.334 M; butanol: 0.088 M. The pattern of effect on membrane fluidity S near the hydrophilic surface was very similar to that on deformability, leading to the significant correlation coefficient observed between deformability and fluidity parameters. Ingolfsson and Anderson, in a study examining various membrane lipid bilayer alterations, found the dependence of the effective concentrations on chain length for a wide range of alcohols [33]. Molecular size- and dose-dependent effects of alcohols on RBC mechanical properties appear to reflect their interactions with lipid bilayer properties, the most notable property being membrane fluidity [34].

The concentration- and chain length-dependent increase of membrane fluidity of the surface layer observed here is in accordance with previous studies on biological membranes [35,36], and with data obtained on simpler model membranes [14,15,33,37]. The impact of alcohols on membrane fluidity might be attributed to the alterations of the free-energy gradient in the lipid bilayer [36]. The insertion of small, mobile amphiphilic molecules into the membrane surface layer affects its compactness by increasing the mobility of lipids and by decreasing their interactions [15]. Pertinent to this, Patra and co-workers have documented that the order parameter of a lipid molecule which is bound to at least one ethanol molecule is lower compared to non-bound lipid [37]. Further, it has been shown that ethanol decreases lipid density in the near-surface layer, but provokes density increase in the middle of the membrane [14,15].

Previous studies on model membranes have shown that at lower concentrations, alcohol molecules are predominantly located in the surface layer of the membrane. However, as alcohol concentration increases, alcohol is pushed towards the deeper membrane layers and inserts into the hydrophobic core [14,15]. The longer the alcohol’s hydrophobic chain the easier it enters the interior of the lipid bilayer [16]. The insertion of alcohol into the middle of the membrane results in augmented interdigitation of lipid chains and increased lipid density [14,15], as shown here by the decreased fluidity of deeper membrane layers. For example, methanol is inserted into the central core region only at high concentrations [37], which is consistent with its weak effects on the fluidity of the core. The potency of short chain alcohols to modify membrane fluidity scales with their bilayer partitioning, which increases with the addition of each methyl group [38,39]. Since the fluidities of the two layers are inter-dependent, the observed gradual decrease of fluidity of deeper membrane layers with increasing concentrations of alcohols might reflect both the decrease of near-surface membrane fluidity as well as the decrease of RBC deformability at higher concentrations. Interestingly, as observed herein, the hemolytic potential of alcohols increases with chain length and partition coefficient [40], thus indicating a link between decreased fluidity of membrane’s core and the initiation of hemolysis [41].

Bilayer fluidity is a key determinant of the function of protein elements within the cell membrane, including ion pump activities, receptor signaling and enzymatic functions [42]. However, data on the correlation of fluidity alterations and functional changes (e.g., the activity of ion pumps) as influenced by alcohols have not always been consistent [35]. It has been suggested that alcohols may affect lipid-protein interactions differently depending on the chain size, or may directly interfere with the protein elements [35]. The association between membrane fluidity and RBC rheological properties has been proposed in relation to specific pathophysiologies such as hypertension [11,13,43], yet fluidity alterations have not always been concordant with RBC deformability data [10,12,44]. The contribution of the membrane skeleton, mainly composed of a spectrin network, as a determinate of rheological behavior is well known [8]. Therefore, the correlated effects of the same alcohols over a similar concentration range on membrane fluidity and RBC deformability may reflect simultaneous changes of both lipid and protein structures. Alcohols may also have more specific effects on protein-protein interactions, via alterations of signaling mechanisms and/or phosphorylation cascades [45], which regulate the associations of the spectrin network with membrane integral proteins [42]. Such alterations have been demonstrated to modulate membrane mechanical properties and RBC deformability [9,46].

It should be noted that alcohols induce morphological alterations, with the degree of echinocytosis increasing with the concentration of ethanol and propanol. A possible cause of echinocytosis could be altered suspending medium osmolality [25], since the presence of alcohol molecules at relatively high concentrations may significantly alter the osmotic properties of the suspending medium. However, RBC membrane permeability (P_d_) for the alcohols utilized in this study (P_d_^meth^ 3.7 x 10^-3^, P_d_^eth^ 2.1 x 10^-3^, P_d_^prop^ 6.5 x 10^-3^, P_d_^but^ ≤ 61 x 10^-3^ cm s^-1^, all at 25°C [47]) are comparable to or even higher than P_d_ for water (5 x 10^-3^ cm.s^-1^, at 25°C [48]). Thus, even in the presence of these alcohols, the extracellular and the intracellular osmolarity are swiftly balanced and there is no net transfer of water across the RBC membrane. Even alcohols with P_d_ slightly lower than P_d_^water^ (e.g., methanol and ethanol), have been shown to cause no net water movement across the RBC membrane [49]. Recently, it has been shown that methanol, ethanol, propanol and butanol may even suppress water diffusion across RBC membrane [50]. The phenomenon was not related to osmolarity changes, but to the dual effects of alcohols on RBC membrane components: water channels have decreased permeability and the lipid bilayer has an increased permeability for water, with the later being related to increased membrane fluidity [51]. The unaltered RBC morphology in methanol and lower concentrations of ethanol also indicates that osmotic gradient were absent.

Echinocytosis may also be the result of preferential incorporation of alcohols into the outer leaflet of the membrane lipid bilayer [25], with the level of influence determined by the molecular size of the alcohol. It should be noted that the alcohols show substantial partition coefficients that increase with molecular size [39]. Using RBC ghosts, Vertessy and Steck have shown that ethanol at concentrations higher than 2% causes cytoskeleton expansion [52], which leads to echinocytosis. Note that the mechanical behavior of RBC is affected by shape transformations [25]. Therefore, deformability data for alcohols that affect RBC morphology should be interpreted with caution, since shape transformation at relatively higher concentrations of each alcohol may also contribute to the biphasic modification of RBC mechanical behavior.

The data presented in this study also imply that membrane/cellular alterations by alcohols did not improve cellular stability as would be expected from observations of improved deformability [53]. It has thus been argued that deformability and stability are two characteristics of RBC membranes, which are determined by different protein-protein interactions [54], thereby providing a possible explanation for the discrepancy between our data on deformability and stability. On the other hand, model membrane studies show that the alcohols used herein cause gradual membrane expansion and decreased thickness with increasing concentrations, and that the effects correlate with the chain length [16], as evidenced by the increased density of the membrane’s core and augmented interdigitation of lipid chains [14,15]. Decreased membrane thickness and tight packing of fatty acid chains could represent another factor involved in alcohol-caused decrease of RBC stability.
